# Nucleotide diversity in the mitochondrial and nuclear compartments of *Chlamydomonas reinhardtii*: investigating the origins of genome architecture

**DOI:** 10.1186/1471-2148-8-156

**Published:** 2008-05-21

**Authors:** David Roy Smith, Robert W Lee

**Affiliations:** 1 Department of Biology, Dalhousie University, Halifax, Nova Scotia, Canada

## Abstract

**Background:**

The magnitude of intronic and intergenic DNA can vary substantially both within and among evolutionary lineages; however, the forces responsible for this disparity in genome compactness are conjectural. One explanation, termed the mutational-burden hypothesis, posits that genome compactness is primarily driven by two nonadaptive processes: mutation and random genetic drift – the effects of which can be discerned by measuring the nucleotide diversity at silent sites (π_silent_), defined as noncoding sites and the synonymous sites of protein-coding regions. The mutational-burden hypothesis holds that π_silent _is negatively correlated to genome compactness. We used the model organism *Chlamydomonas reinhardtii*, which has a streamlined, coding-dense mitochondrial genome and an noncompact, intron-rich nuclear genome, to investigate the mutational-burden hypothesis. For measuring π_silent _we sequenced the complete mitochondrial genome and portions of 7 nuclear genes from 7 geographical isolates of *C. reinhardtii*.

**Results:**

We found significantly more nucleotide diversity in the nuclear compartment of *C. reinhardtii *than in the mitochondrial compartment: net values of π_silent _for the nuclear and mitochondrial genomes were 32 × 10^-3 ^and 8.5 × 10^-3^, respectively; and when insertions and deletions (indels) are factored in, these values become 49 × 10^-3 ^for the nuclear DNA and 11 × 10^-3 ^for the mitochondrial DNA (mtDNA). Furthermore, our investigations of *C. reinhardtii *revealed 4 previously undiscovered mitochondrial introns, one of which contains a fragment of the large-subunit (LSU) rRNA gene and another of which is found in a region of the LSU-rRNA gene not previously reported (for any taxon) to contain introns.

**Conclusion:**

At first glance our results are in opposition to the mutational-burden hypothesis: π_silent _was approximately 4 times greater in the nuclear compartment of *C. reinhardtii *relative to the mitochondrial compartment. However, when we consider the encumbrance of noncoding DNA in each of these *C. reinhardtii *compartments, we conclude that introns in the mtDNA impose a greater burden than those in the nuclear DNA and suggest that the same may be true for the intergenic regions. Overall, we cannot reject the mutational-burden hypothesis and feel that more data on nucleotide diversity from green algae and other protists are needed.

## Background

Genomic sequence data from the three domains of life have revealed a prodigious range of genome compactnesses; however, our knowledge of the processes responsible for this gamut of genomic architectures is contentious. One explanation is the mutational-burden hypothesis [1-4], which posits that genome compactness (defined by the proportion of intronic and intergenic DNA) is primarily driven by nonadaptive processes: namely, mutation and random genetic drift. The mutational-burden hypothesis asserts that noncoding DNA (i.e., intronic and intergenic DNA) is a genetic liability because it is a target for deleterious and potentially lethal mutations, such as mutations affecting sequences involved with intron splicing and gene regulation. The hypothesis maintains that species with large effective population sizes (*N*_*e*_) are more efficient at purging, or preventing the proliferation of, hazardous-noncoding DNA because they experience less random genetic drift and thereby increase the efficacy of natural selection. The mutational-burden hypothesis holds that the product of *N*_*e *_and the mutation rate (μ) drives genome compactness; consequently, species whose genomes are coding rich should have a higher *N*_*e*_μ than those whose genomes carry a surfeit of intronic and intergenic DNA.

Insights into *N*_*e *_and μ can be acquired by measuring the nucleotide diversity at silent sites (π_silent_), which are defined as noncoding sites and synonymous sites within protein-coding DNA. Since, for a diploid population at mutation-drift equilibrium, the rate at which new variation is introduced to a neutral-nucleotide site in two randomly compared alleles is equivalent to 2μ (twice the mutation rate), and the rate at which variation is lost from a neutral site is 1/2*N*_*e*_, then the average number of nucleotide differences per neutral site is equivalent to the ratio of these two rates: 4*N*_*e*_μ. Because silent sites are typically regarded as among the most neutrally evolving positions in a genome, measures of π_silent _can provide an estimate of 4*N*_*e*_μ. This formula can be simplified by substituting *N*_*g*_, the effective number of genes per locus in a population, for *N*_*e*_, giving a final equation of π_silent _= 2*N*_*g*_μ, where *N*_*g *_is equal to *N*_*e *_for nuclear genes of haploid species and about one-half *N*_*e *_for uniparentally-transmitted organelle genes [5]. Uniparentally-transmitted organelle genomes (mitochondrial or chloroplast) are generally considered haploid, despite being present in multiple copies per cell, because heteroplasmy (the existence of more than one organelle-genome haplotype in the same individual) is rare. Moreover, in this instance, *N*_*g *_is reduced further by the fact that during sexual reproduction only one of the parental sexual types transmits organelle genes to the next generation.

Large-scale studies have found a positive correlation between π_silent _and genome compactness: in the compact genomes of prokaryotes π_silent _tends to be > 50 × 10^-3^; in the more bloated nuclear genomes of invertebrates and land plants it is in the range of 3 × 10^-3 ^to 15 × 10^-3^; and in the nuclear genomes of vertebrates, where noncoding DNA predominates, it appears to lie between 2 × 10^-3 ^and 4 × 10^-3 ^[1]. There is evidence that this trend is also found in organelle genomes: comparative studies of nucleotide diversity in mitochondrial DNA (mtDNA) indicate that in the diminutive, coding-dense mitochondrial genomes of mammals π_silent _is around 40 × 10^-3^, whereas in the expanded mitochondrial genomes of land plants it is estimated to be < 0.4 × 10^-3 ^[3]. This disparity in π_silent _between land-plant and mammalian mitochondrial genomes is believed to be a reflection of the high mutation rates in mammalian mtDNA and the low mutation rates typically found in land-plant mtDNA. Mutation rates have also been invoked to explain why, despite similar proposed values of *N*_*g*_, the mitochondrial and nuclear genomes of mammals have opposite coding densities – in mammals estimates of μ for mtDNA are roughly 30 times those for nuclear DNA [3]. Although the relationship between π_silent _and genome architecture is intriguing, the empirical data from which these correlations were derived are limited to a relatively small number of taxa and are generally skewed towards multicellular animals, with an overall lack of data for unicellular eukaryotes, especially green algae.

The model organism *Chlamydomonas reinhardtii*, a unicellular green alga of the chlorophycean class, is an excellent system for studying the evolution of genome compactness because it has a large, intron-rich nuclear genome and a small, compact mitochondrial genome, yet both genomes appear to have a similar mutation rate [6,7]. The nuclear genome of *C. reinhardtii*, which has been sequenced to 95% completion, is approximately 121 megabases (Mb), with about 17% of the nucleotides coding for proteins and structural RNAs [8]. Furthermore, the genome has an abundance of introns (~7 per protein-coding gene), and the average intron length is longer than that of many eukaryotes and is more similar to multicellular organisms than to protists. In contrast, the mitochondrial genome of the standard laboratory strains of *C. reinhardtii *(derived from the Ebersold-Levine line) is streamlined, having a size of 15.8 kb and containing only 13 genes [9-11]. Moreover, at 82% coding it is one of the most compact mitochondrial genomes available from green algae (for a compilation see [12]), and although the mtDNA of one geographical isolate of *C. reinhardtii *(CC-1373) has an optional intron in *cob *[13] it is still > 75% coding; this strain is often referred to as *Chlamydomonas smithii *but is in fact a member of the *C. reinhardtii *species [14].

According to the mutational-burden hypothesis, we would expect *C. reinhardtii *to have a high degree of silent-site nucleotide diversity in its mitochondrial genome (reflecting a large 2*N*_*g*_μ) and a low degree of silent-site nucleotide diversity in its nuclear genome (reflecting a small 2*N*_*g*_μ). To test this hypothesis and to investigate the correlation of 2*N*_*g*_μ with genome compactness, we measured π_silent _in the mitochondrial and nuclear compartments from various geographical isolates of *C. reinhardtii*.

## Results

### Strains and their genetic loci

**Figure 1:**
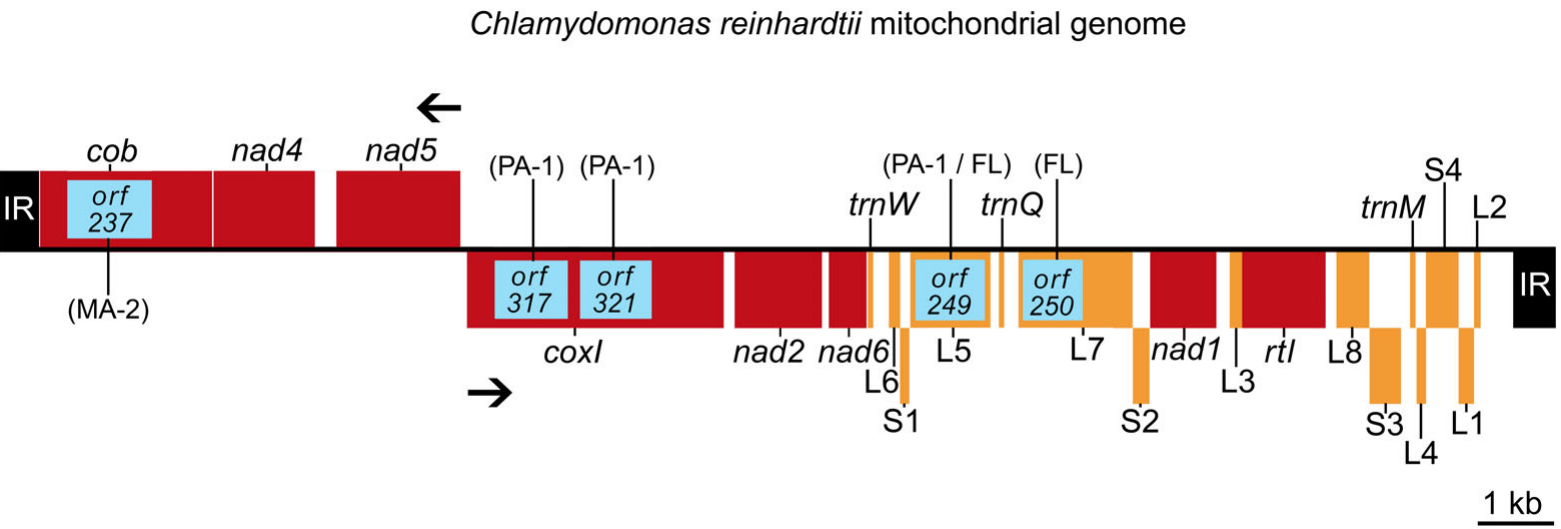
**Genetic map of the *Chlamydomonas reinhardtii *mitochondrial genome, including all currently identified optional introns**. Protein-coding regions and regions encoding structural RNAs are red and orange, respectively. S1–S4 represent the small-subunit rRNA-coding modules; L1–L8 represent the large-subunit rRNA-coding modules. The terminal inverted repeats (IR) are black. Intronic regions and their open reading frames are boxed in blue inside their associated genes. The *C. reinhardtii *strains (Table 1) in which the different introns occur are labelled in parentheses. Solid arrows denote the transcriptional polarities. Note: due to the presence/absence of introns among the different strains, the size of the *C. reinhardtii *mitochondrial genome can vary from 15,782 nt to 18,990 nt.

**Figure 2:**
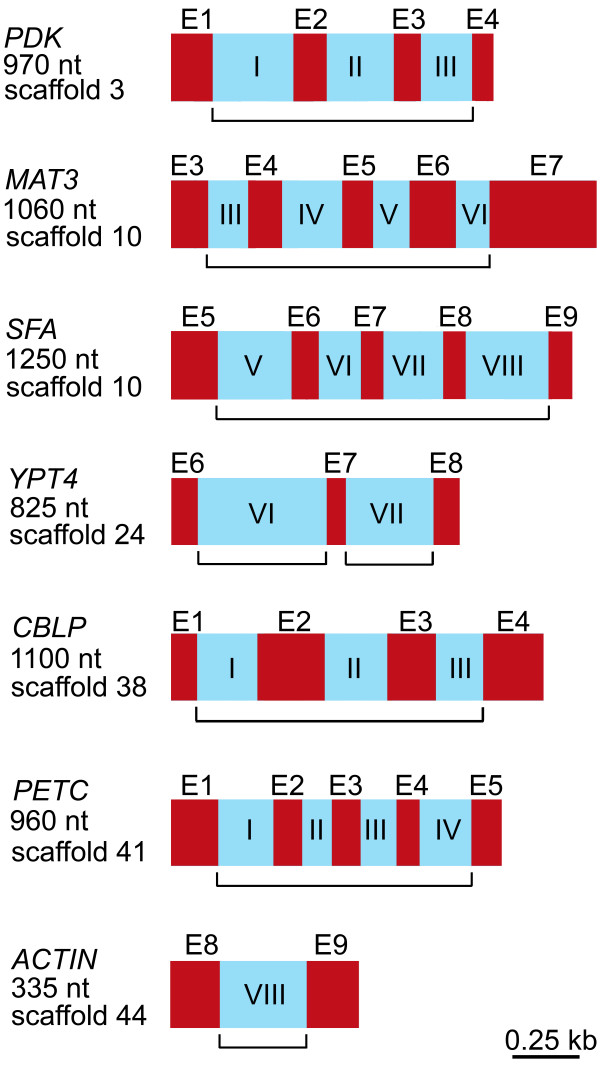
**Partial genetic maps of the 7 *Chlamydomonas reinhardtii *nuclear-encoded genes employed in the analysis**. The bracketed segment beneath each map represents the region that was PCR amplified. Left of each map is the name of the gene, the approximate size of the region that was PCR amplified, and the location of the gene within the *C. reinhardtii *nuclear genome – locations are based on the *C. reinhardtii *draft nuclear genome sequence version 3.0 [8]. Exons are red; they are labelled with an "E" and a number denoting their position within the gene. Introns are blue and are labelled with a roman numeral denoting their location within the gene. Note: each of these genes is present only once in the *C. reinhardtii *nuclear genome.

Seven geographical isolates of *C. reinhardtii *were employed in this study; their strain numbers, mating types, origins of isolation, and strain abbreviations are presented in Table 1. To access levels of genetic diversity we sequenced the complete mitochondrial genome and portions of 7 single-copy nuclear genes from each of the 7 isolates. A genetic map of the *C. reinhardtii *mitochondrial genome is shown in Figure 1, and partial genetic maps of the 7 nuclear genes are shown in Figure 2. We sequenced the entire mtDNA in order to employ both intergenic regions and synonymous sites in our calculations of π_silent _– previous studies on genetic diversity in mitochondrial genomes, due to a paucity of intraspecific sequence data, have tended to use only synonymous sites for estimating π_silent_. Moreover, whole mtDNA sequences from *C. reinhardtii *allow for the comparison of synonymous-site nucleotide diversity (π_syn_) in the standard mitochondrial protein-coding genes to that of *rtl*, a mitochondrial open reading frame (ORF) in the *C. reinhardtii *mtDNA coding for a putative reverse-transcriptase-like protein [15]. It has been suggested that synonymous sites in *rtl *are under less selective constraints than those of the standard mtDNA protein-coding genes and that they may be more appropriate for estimating the neutral mutation rate in the mitochondrial compartment [7]. For the nuclear loci, we sequenced mostly introns rather than exons because it is believed that in the *C. reinhardtii *nuclear genome intronic sites are more neutrally evolving than synonymous sites and may give more reliable estimates of the neutral mutation rate [6]. Sequences for two of the nuclear loci from the 7 isolates have been previously reported [16-18] allowing us to confirm both our strain assignations and sequencing methods.

**Table 1:** *Chlamydomonas reinhardtii *strains used in this study.

Strain	Mating Type	Strain Synonym	Geographical Origin (USA)	**Abbreviation **^a^	Reference
CC-277	*mt*^+^	*cw15*	Amherst, Massachusetts	MA-1	Harris 1989 [20]
CC-1373	*mt*^+^	*C. smithii*	South Deerfield, Massachusetts	MA-2	Hoshaw and Ettl 1966 [41]
CC-1952	*mt*^-^	*C. grossii*	Plymouth, Minnesota	MN	Gross et al. 1988 [42]
CC-2342	*mt*^-^	Jarvik 6	Pittsburgh, Pennsylvania	PA-1	Spanier *et al*. 1992 [43]
CC-2344	*mt*^+^	Jarvik 356	Malvern, Pennsylvania	PA-2	Spanier *et al*. 1992 [43]
CC-2931	*mt*^-^	Harris 6	Durham, North Carolina	NC	Harris 1989 [20]
CC-2343	*mt*^+^	Jarvik 124	Melbourne, Florida	FL	Spanier *et al*. 1992 [43]

^a ^Strain abbreviations are based on the USA state from which the strains were isolated.

We were able to obtain the complete mtDNA sequence from an 8^th ^strain of *C. reinhardtii *(CC-503) by collecting and assembling mtDNA sequences that were generated from the *C. reinhardtii *nuclear-genome sequencing project [8,19]. Both *C. reinhardtii *CC-503 and *C. reinhardtii *CC-277 (one of the 7 isolates described in Table 1) are cell-wall-less mutants recovered from the same "Ebersold-Levine" wild-type background of *C. reinhardtii*, but they have been separated for at least 35 years [20]. The mtDNA sequence of *C. reinhardtii *CC-503 is identical to that of *C. reinhardtii *CC-277; and when we downloaded the sequences of the 7 nuclear loci for *C. reinhardtii *CC-503, they too were identical to those of *C. reinhardtii *CC-277. Therefore, for the purpose of this study we will be considering *C. reinhardtii *MA-1 as synonymous with *C. reinhardtii *CC-277 and CC-503.

Prior to this study a complete mtDNA sequence for *C. reinhardtii *was already available (Genbank accession number NC_001638); this sequence, which resulted from the accumulated efforts of multiple parties, mostly came from *C. reinhardtii *CC-277, or in some cases from strains having the same genetic background as *C. reinhardtii *CC-277. The mtDNA sequence of *C. reinhardtii *CC-277 presented here differs at 46 positions relative to NC_001638; because 44 of these 46 differences are also present in the mtDNA of the other six *C. reinhardtii *isolates described here and because the *C. reinhardtii *CC-277 mtDNA sequence from this study was shown to be identical to the *C. reinhardtii *CC-503 mitochondrial genome, we feel that our version of the *C. reinhardtii *CC-277 mtDNA is currently the most accurate and that the discrepancies between our sequence and NC_001638 are the result of sequencing errors in the latter.

It is important to note that our annotation of the *C. reinhardtii *mitochondrial genome (Figure 1) does not contain the so-called rRNA-coding modules L2b and L3a. In previous studies each these modules was presumed to code for a non-core region of the large-subunit (LSU) rRNA [21]. However, because sequence homologs of L2b and L3a have not been identified in the mtDNA of close relatives to *C. reinhardtii *[7,22,23], or in any other genome, we have classified these regions as intergenic DNA, and have treated them as such for all genetic analyses.

### Nucleotide diversity

Summary statistics of the nucleotide diversity in *C. reinhardtii *are shown in Table 2. Two measures of nucleotide diversity were used to calculate variation within the *C. reinhardtii *mitochondrial and nuclear genomes: π, which is the average number of pair-wise nucleotide differences per site between sequences in a sample [24], and θ_W_, which is based on the number of polymorphic sites in a sample of sequences but is independent of their frequency [25]. With respect to both measures, the nuclear compartment shows significantly more silent-site nucleotide diversity than the mitochondrial compartment: net values of π_silent _for the nuclear DNA and mtDNA were 31.96 × 10^-3 ^and 8.54 × 10^-3^, respectively. Net values of θ_W _at silent sites are slightly higher at 33.02 × 10^-3 ^for the nuclear compartment and 9.18 10^-3 ^for the mitochondrial compartment. In all cases, silent sites in the various nuclear loci show more diversity than the silent sites in the mitochondrial compartment. The only exception to this is the nuclear gene *CBLP*, which has less synonymous-site diversity (π_syn _= 2.77 × 10^-3^) than that of the mitochondrial protein-coding regions. Within the mitochondrial compartment, diversity at intergenic and synonymous sites is similar (8.92 × 10^-3 ^vs. 8.52 × 10^-3^), as is the diversity of protein-coding regions and regions coding for structural RNAs (2.06 × 10^-3 ^vs. 2.42 × 10^-3^). The mitochondrial gene *rtl*, which encodes a putative reverse transcriptase, shows more diversity than the other mitochondrial protein-coding genes when all 3 codon sites are considered (3.07 × 10^-3 ^vs. 2.06 × 10^-3^) and slightly less diversity when looking only at synonymous sites (7.88 × 10^-3 ^vs. 8.52 × 10^-3^); however, it is unlikely that these observations are statistically significant.

**Table 2:** Nucleotide diversity in the mitochondrial and nuclear compartments of *Chlamydomonas reinhardtii*.

			# of sites	*S*	# of Indels^g ^(length nt)	π × 10^-3 ^(SD × 10^-3^)	θ_W _× 10^-3 ^(SD × 10^-3^	π_syn _× 10^-3^	π_ns _× 10^-3^	Tajima's D test (P value)
**mtDNA**	Complete genome^a^		15280	134	15 (36)	3.35 (0.68)	3.66 (0.31)	---	---	-0.49 (> 0.10)
	Protein-coding^b^		8154	44	1 (6)	2.06 (0.43)	2.20 (0.33)	8.52	0	-0.38 (> 0.10)
	structural RNA genes^c^		3502	23	4 (4)	2.42 (0.42)	2.68 (0.56)	---	---	-0.55 (> 0.10)
	*rtl*		1118	9	1 (3)	3.07 (0.87)	3.29 (1.10)	7.88	1.89	-0.35 (> 0.10)
	Intergenic^d^		2434	58	9 (23)	8.92 (1.88)	9.73 (1.28)	---	---	-0.48 (> 0.10)
	Silent sites^e^		5152	99	12 (26)	8.54 (1.03)	9.18 (0.65)	---	---	---
**Nuclear DNA**	Intronic (overall)		4294	359	47 (216)	33.50 (3.15)	33.81 (1.79)	---	---	-0.16 (> 0.10)
	Silent sites^f^		4824	381	50 (219)	31.96 (3.03)	33.02 (1.88)	---	---	---
	Exonic (overall)		1614	25	1 (9)	6.02 (0.99)	6.58 (1.29)	19.57	1.42	-0.48 (> 0.10)
	Exonic (by gene)	*CBLP*	420	1	0	0.68 (0.47)	0.97 (0.97)	2.77	0	-1.00 (> 0.10)
		*PETC*	300	2	0	2.54 (0.78)	2.72 (1.92)	9.69	0	-0.27 (> 0.10)
		*PDK*	222	3	0	6.86 (1.88)	5.52 (3.18)	26.35	0	1.10 (> 0.10)
		*MAT3*	408	10	1 (9)	10.50 (2.70)	10.00 (3.16)	24.37	4.77	0.27 (> 0.10)
		*SFA*	264	9	0	9.74 (2.80)	13.91 (4.64)	41.14	0	-1.59 (> 0.10)
	Intronic (by gene)	*CBLP*	578	77	8 (75)	58.30 (7.41)	54.30 (6.20)	---	---	0.26 (> 0.10)
		*PETC*	621	43	3 (5)	30.21 (6.07)	28.26 (4.31)	---	---	0.39 (> 0.10)
		*PDK*	691	37	8 (42)	23.22 (5.58)	21.86 (3.59)	---	---	0.20 (> 0.10)
		*MAT3*	560	30	4 (25)	21.60 (3.03)	21.87 (3.99)	---	---	-0.07 (> 0.10)
		*SFA*	847	98	12 (15)	43.57 (4.53)	48.67 (4.77)	---	---	-0.61 (> 0.10)
		*YPT4*	790	47	9 (39)	22.66 (4.50)	24.28 (3.54)	---	---	-0.38 (> 0.10)
		*ACTIN*	207	27	3 (15)	53.37 (8.18)	53.37 (10.2)	---	---	0.01 (> 0.10)

Note--*S*, number of segregating sites; Indels, insertion and deletions; π, nucleotide diversity; θ_W_, Theta (per-site) from Watterson estimator; π_syn_, nucleotide diversity at synonymous sites; π_ns_, nucleotide diversity at nonsynonymous sites; SD, standard deviation.

^a ^Includes only 1 telomere.

^b ^Includes all protein-coding genes excluding *rtl*.

^c ^Includes rRNA- and tRNA-coding regions.

^d ^Includes intergenic regions and 1 telomere.

^e ^Includes synonymous sites, intergenic regions, and 1 telomere.

^f ^Includes synonymous sites and intronic regions.

^g ^Nucleotide diversity does not include indels. Consecutive indel sites are counted as single event. Indel length refers to the sum of all indels.

### Insertions and deletions

For both the nuclear and mitochondrial compartments, insertions and deletions (indels) represent a large proportion of the observed polymorphisms (Table 2). In our alignments of the nuclear loci from the 7 different strains of *C. reinhardtii*, 36% of mismatched nucleotides result from indels. The nuclear indels range from 1–31 nucleotides (nt) in length and have an average size of 4.5 nt. In the mitochondrial compartment indels represent 20% of the mismatched nucleotides. The mitochondrial indels range from 1–6 nt in length and have an average size of 2.5 nt. It is important to note that our estimates of nucleotide diversity shown in Table 2 are derived from sites in the alignment where all seven strains of *C. reinhardtii *have a nucleotide; therefore, sites corresponding to indels were removed from the alignment. If our methods for calculating π are modified to include indels (by counting each gap in the alignment as a nucleotide change) the overall values of π_silent _in the nuclear and mitochondrial compartments become 49.27 × 10^-3 ^(± 4.89 × 10^-3^) and 10.93 × 10^-3 ^(± 1.96 × 10^-3^), respectively.

### Testing for neutrality

Two statistical tests were performed on the mitochondrial and nuclear datasets to examine for traces of selection: Tajima's *D*-test, which compares the average number of nucleotide differences between pairs of sequences to the total number of segregating sites [24], and the McDonald-Kreitman test, which compares the ratio of nonsynonymous to synonymous differences observed within a species to that observed between species [26]. Tajima's *D *is slightly negative in all cases pertaining to the mitochondrial compartment and in most cases pertaining to the nuclear compartment, but it is slightly positive for a few of the nuclear loci (the exons of *MAT3 *and *PDK*, and the introns of *CBLP, PETC, PDK*, and *ACTIN*) (Table 2). In no case is Tajima's *D*-test statistically significant. The McDonald-Kreitman test was performed by comparing the ratio of nonsynonymous to synonymous polymorphisms within *C. reinhardtii *to the ratio of nonsynonymous to synonymous fixed differences between *C. reinhardtii *and *Chlamydomonas incerta *(one of the closest known non-interfertile relatives of *C. reinhardtii *[27]) (Table 3) – this was done for all of the protein-coding regions surveyed in this study. Overall, no significant departures from neutral expectations were detected for any of the mitochondrial or nuclear loci, and in no case is the McDonald-Kreitman test statistically significant.

**Table 3:** McDonald-Kreitman test comparing the ratio of nonsynonymous to synonymous differences within *Chlamydomonas reinhardtii *to that found between *C. reinhardtii *and *Chlamydomonas incerta*.

			Polymorphisms within *C. reinhardtii*	Substitutions between *C. reinhardtii *and *C. incerta*	*NI*	*G*	*P*
**mtDNA**	Protein-coding^a^	Nonsynonymous	3	61	0.653	0.533	0.465
		Synonymous	36	478			
	*rtl*	Nonsynonymous	5	111	0.746	0.185	0.667
		Synonymous	4	119			
**Nuclear DNA**	Exonic (overall)^b^	Nonsynonymous	1	18	0.159	4.837	0.027
		Synonymous	21	60			
	*CBLP*	Nonsynonymous	0	0	undef	undef	undef
		Synonymous	1	1			
	*PETC*	Nonsynonymous	0	3	0.000	undef	undef
		Synonymous	2	4			
	*PDK*	Nonsynonymous	0	2	0.000	undef	undef
		Synonymous	3	18			
	*MAT3*	Nonsynonymous	1	9	0.677	1.081	0.298
		Synonymous	6	18			
	*SFA*	Nonsynonymous	0	4	0.000	undef	undef
		Synonymous	9	19			

Note--*NI*, neutrality index (ratio of nonsynonymous to synonymous polymorphisms within *C. reinhardtii *compared to the ratio of nonsynonymous to synonymous fixed differences between *C. reinhardtii *and *C. incerta*); *G*, G-test of independence (determines if the proportion of nonsynonymous substitutions is independent of whether the substitutions are fixed or polymorphic); *P*, probability of G-test; undef, undefined. *C. incerta *data came from [6] and [7]. Note: in no case was the McDonald-Kreitman test statistically significant.

^a ^Includes all protein coding genes excluding *rtl*.

^b ^Concatenated exons from all 5 nuclear genes.

### Mitochondrial introns

**Figure 3:**
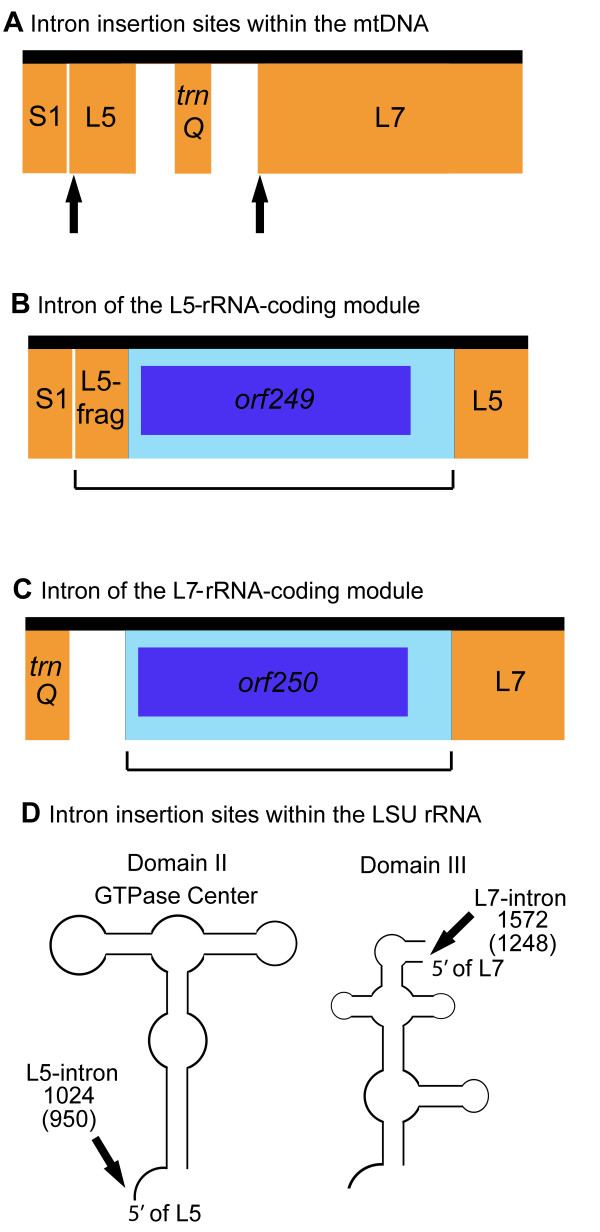
**Schema of the introns in the L5- and L7-rRNA-coding modules**. The vertical arrows in **A **show the intron insertion sites within the *C. reinhardtii *mtDNA. **B **and **C **depict the introns in the L5- and L7-rRNA-coding modules, respectively; rRNA-coding regions are orange; introns are light blue; intronic open reading frames are boxed in dark blue within their respective introns; L5-frag refers to a duplicated segment of the L5-rRNA-coding module (the first 35 nt of the module are duplicated); bracketed portions of the map represent regions that were shown to be spliced-out in mature transcripts. **D **depicts the intron insertion sites in the context of the large subunit (LSU) ribosomal RNA sequence of *C. reinhardtii*; arrows point to the region where the introns are inserted; numbers above the arrows denote the position of the residue that immediately precedes the insertion site: un-bracketed numbers correspond to the residue in the 23S rRNA gene of *Escherichia coli *[44] and bracketed numbers correspond to the residue in the LSU-rRNA secondary-structure model of Boer and Gray [21]. Note: the *C. reinhardtii *strains in which these introns occur are shown in Figure 1.

Three of the *C. reinhardtii *strains (PA-1, MA-2, and FL) have introns in their mtDNA (Figure 1). *C. reinhardtii *MA-2 has a single intron, inserted into *cob*; *C. reinhardtii *FL has 2 introns, one in the L5-rRNA-coding module (the L5-intron) and one in the L7-rRNA-coding module (the L7-intron); and *C. reinhardtii *PA-2 has 3 introns, two in *cox1 *and the L5-intron (note: the DNA sequence of the L5-intron in *C. reinhardtii *PA-2 is identical to that of *C. reinhardtii *FL). Of these introns only that of *cob *in *C. reinhardtii *MA-2 has been previously described [13]. Like the intron of *cob *in *C. reinhardtii *MA-2, each of the 4 introns presented here has an ORF for which the deduced amino acid sequence shows similarity to a LAGLIDADG-type endonuclease. RT-PCR experiments confirm that all five introns, including their ORFs, are spliced-out in mature transcripts. Secondary-structure modelling suggests that the two introns in *cox1 *are group I introns belonging to subgroup D. Our analyses of the L5- and L7-introns suggest that they lack the core sequence and potential secondary structure necessary to be classified as either group I or group II introns; thus, at the present time they are considered highly-degenerate "unclassified" introns. A 35-nt duplicated portion of the L5-rRNA-coding module is found within the 5' end of the L5-intron; RT-PCR experiments validate that this segment is in fact a component of the intron. The insertion sites of the L5- and L7-introns within the *C. reinhardtii *mtDNA and the nature of the repeat found within the L5-intron are described in Figure 3A, B, and 3C, respectively. The insertion sites of the L5- and L7-introns in context to the *C. reinhardtii *LSU rRNA sequence are shown in Figure 3D.

## Discussion

### Accounting for the differences in π_silent_

Before we interpret our data on nucleotide diversity in relation to the mutational-burden hypothesis, let us first try to account for the values of π_silent _that we observed. Overall, in *C. reinhardtii *we found 3.7-fold more nucleotide diversity at silent sites in the nuclear compartment than in the mitochondrial compartment; and when indels are taken into consideration, π_silent _appears to be 4.5 times greater in the nuclear DNA compared to the mtDNA. Assuming that π_silent _is equal to 2*N*_*g*_μ, we can discuss our findings on nucleotide diversity in relation to μ and *N*_*g*_.

In a recent study that compared silent-site substitution rates in the mitochondrial and nuclear genomes between *C. reinhardtii *and *C. incerta*, it was concluded that the mutation rate in the nuclear compartment of these taxa is approximately the same as that in the mitochondrial compartment [6,7]. If is similar in both the nuclear DNA and mtDNA of *C. reinhardtii*, then it appears that differences in *N*_*g *_would have to explain the disparity in nucleotide diversity that we observe between these genomes.

In order to arrive at the values of π_silent _observed in this study, *N*_*g *_would have to be higher for the nuclear genome than for the mitochondrial genome (again, assuming equal mutation rates); there are a few reasons why this might be the case. For the haploid alga *C. reinhardtii*, nuclear genes are inherited biparentally and mitochondrial genes are inherited uniparentally [28]. As mentioned earlier, this is thought to reduce *N*_*g *_in the mitochondrial compartment by approximately one-half relative to that in the nucleus [5]. Thus, for *C. reinhardtii *we might expect a value of π_silent _in the mitochondrial compartment to be around one-half of what is observed in the nuclear compartment. Uniparental inheritance also implies that the mtDNA of *C. reinhardtii *has less opportunity for recombination than the nuclear DNA, which may make the mitochondrial genome as a whole more susceptible to the effects of selective sweeps and purifying selection, both of which can reduce *N*_*g*_, resulting in an even smaller than expected value of π_silent _in the mitochondrial compartment [29,30]. One way to detect the influences of selection is with the McDonald-Kreitman test, where positive selection is inferred if the test returns a value for the neutrality index (NI) < 1, and purifying selection is indicated by NI > 1 [29-31]. Although the results of the MacDonald-Kreitman test for *C. reinhardtii *versus *C. incerta *showed no significant departure from neutral expectations, values of NI were < 1 for *rtl *and the concatenated sequence of the standard mitochondrial protein-coding regions (Table 3), which might be an indication of positive selection. Moreover, Tajima's *D *test returned negative values for all of the mtDNA regions that were examined (Table 2) – negative values of Tajima's *D *test can be an indication of a recent selective sweep of a linked mutation [32] – but again these findings were not statistically significant.

A further consideration is that the mutation rate in the *C. reinhardtii *mitochondrial compartment may be slightly lower than that in the nuclear compartment. When Popescu and Lee [7] estimated μ to be similar for both the nuclear and mitochondrial genomes of *C. reinhardtii*, they used the synonymous substitution rate in *rtl *(which was about double that of the standard mitochondrial protein-coding regions) as an estimate of the neutral mutation rate in the mitochondrial compartment. In contrast, we found π_syn _in *rtl *to be similar to that of the standard mitochondrial protein-coding regions (7.88 × 10^-3 ^vs. 8.52 × 10^-3^), and although this could be an artefact of a small sample size, it might suggest that Popescu and Lee overestimated μ in the *C. reinhardtii *mtDNA.

### Π_silent _in relation to previous studies on *C. reinhardtii *and other unicellular eukaryotes

Our estimates of π_silent _in the nuclear and mitochondrial genomes of *C. reinhardtii *are approximately 32 × 10^-3 ^and 8.5 × 10^-3^, respectively; and with indels factored in, these values become ~50 × 10^-3 ^for the nuclear DNA and about 11 × 10^-3 ^for the mtDNA. The only other estimates of nucleotide diversity in the *C. reinhardtii *nuclear and mitochondrial genomes that we could find were that of Lynch and Connery [1], who, using 11 kb of mostly noncoding nuclear DNA from *C. reinhardtii *strains MA-1 and MN, found π_silent _in the nuclear compartment to be ~40 × 10^-3 ^(they estimated *N*_*e*_μ to be ~20 × 10^-3^), which is in agreement with our observations. With respect to other green algae, we are unaware of any reported estimates of within-population silent-site variation in either nuclear or mitochondrial genomes.

There is an overall lack of data on π_silent _for unicellular species; however, the data that are available are relatively consistent with our results for *C. reinhardtii*. Among unicellular fungi, average values of π_silent _in the nuclear and mitochondrial compartments (based on data for 3 different species) were estimated to be 50 × 10^-3 ^and 12 × 10^-3^, respectively [1,3], which is comparable to what we observed in *C. reinhardtii*. Furthermore, the genera *Paramecium *and *Trypanosoma *appear to have ratios of π_silent(mitochondrion)_/π_silent(nucleus) _of approximately 0.4 [3], which is within the range of our estimates for *C. reinhardtii *(0.2–0.25). All studies deriving measures of π_silent _from the mitochondrial genome of unicellular eukaryotes have used 3 or fewer loci for their estimates and have tended to focus only on synonymous sites, which makes our analysis one of the most comprehensive for any mitochondrial genome from a unicellular eukaryote to date.

It is also of interest to compare the nucleotide diversity of *C. reinhardtii *to that of multicellular species. Our estimation of nucleotide diversity in the nuclear genome of *C. reinhardtii *is much greater than that observed for animals: ~9 times larger than the average estimate for mammals (3.6 × 10^-3^), and 2 times greater than the average for invertebrates, which is believed to be < 14.8 × 10^-3 ^[1]. Our approximation of π_silent _for the mitochondrial genome of *C. reinhardtii *is much smaller than that of animals: 0.2 times the average for mammals (40 × 10^-3^) and 0.1–0.6 times the average for invertebrates (11 × 10^-3^– 67 × 10^-3^) [2]. In comparison to land plants, *C. reinhardtii *has twice the amount of silent-site nucleotide variation in its nuclear genome (π_silent _in land plants is estimated to be ~15 × 10–3), and although we are unaware of any reliable assessments of π_silent _in land plant mtDNA, it is purported to be < 0.4 × 10^-3 ^[3], which is less than 0.05 times what we observed for the *C. reinhardtii *mtDNA.

### Testing the mutational-burden hypothesis

At first glance, our estimates of nucleotide diversity in the mitochondrial and nuclear genomes of *C. reinhardtii *appear contrary to what would be expected under the mutational-burden hypothesis. We found π_silent _to be ~4 times greater in the nuclear compartment than in the mitochondrial compartment, but based on the streamlined nature of the *C. reinhardtii *mitochondrial genome in relation to its noncompact nuclear genome, one might expect the mutational-burden hypothesis to predict a greater value of π_silent _for the mtDNA. However, before we conclude that our findings are in opposition to the mutation-burden hypothesis, we must first consider what the actual "encumbrance" of noncoding DNA is for the mitochondrial and nuclear genomes in *C. reinhardtii*.

As described earlier, the basic premise of the mutation-burden hypothesis is that noncoding DNA magnifies the target site for deleterious mutations, thereby, increasing the susceptibility of a genome to degenerative changes. The mutational disadvantage of noncoding DNA, therefore, is critically dependent on: 1) the number of nucleotides that are associated with gene function (*n*), and 2) the per-nucleotide mutation rate (μ). These two terms can be combined to define the overall mutational disadvantage (*s*), where *s *= *n*μ [3,33]. It is predicted that the threshold in a genome below which noncoding DNA can proliferate is 2*N*_*g*_*s *< 1, or alternatively 2*N*_*g*_μ < 1/*n *[3,33]. Although it is difficult to estimate *n *for intergenic regions, values of *n *for intronic regions can be predicted with reasonable confidence. For the spliceosomal introns of eukaryotic genomes, *n *is believed to be around 25 [33], which gives a threshold for intron colonization in the nuclear compartment of 2*N*_*g*_μ < 0.04. Because mitochondrial introns are self-splicing and do not rely on a spliceosome for excision they have more nucleotides that are critical for proper splicing; thus, we can conservatively say that for mitochondrial introns *n *is between 75–100 (see [34] for a review on mitochondrial-intron folding), giving a threshold for intron proliferation in the mitochondrial compartment of ~2*N*_*g*_μ < 0.01. Based on the mutational-burden hypothesis, our estimates of 2*N*_*g*_μ (i.e. π_silent_) in the nuclear and mitochondrial compartments of *C. reinhardtii*, whether including or excluding the influence of indels, lie too close to the predicted thresholds for intron proliferation in these genomes to accurately forecast intron abundance.

We do not know what the encumbrance of intergenic regions is for either the nuclear or the mitochondrial genome of *C. reinhardtii*, but if it is (or was at some point in the past) substantially higher for the mtDNA than the nuclear DNA, it would indicate that the mitochondrial compartment is a less permissive environment for the proliferation of intergenic DNA. And although this is highly speculative, there is one reason why this might be the case. In the *C. reinhardtii *mitochondrial genome, mature-RNA transcripts are generated by precise endonucleolytic cleavage of long polycistronic precursor-messenger RNAs; it is believed that these immature transcripts are cleaved in regions of the RNA corresponding to intergenic sites in the mitochondrial genome, and that processing is critically dependent on the primary sequence and the secondary structure of these regions [9]. This implies that the intergenic sites in the *C. reinhardtii *mitochondrial genome may have a large mutational burden associated with them, perhaps large enough to impose a barrier on the amplitude of intergenic mtDNA. Moreover, the polycistronic nature of the *C. reinhardtii *mitochondrial transcripts suggest that the regulatory elements within the intergenic mtDNA carry the increased burden of being responsible for many genes – a burden not typically associated with the monocystronic gene regulation of nuclear DNA [35].

If the burden of intronic and intergenic DNA is higher in the mtDNA of green algae and other protists, then we might expect to find very low values of π_silent _in the mitochondrial genomes of species from these groups that have an abundance of intronic and intergenic sequences – for examples see references [12,36,37].

It is worth noting that the *C. reinhardtii *chloroplast genome when compared to its mitochondrial counterpart has a similarly low density of introns but a substantially greater proportion of intergenic DNA [38]. Using 1500 nt of chloroplast DNA (composed of the *pet*A gene and a single intergenic region) we found no nucleotide polymorphisms in any of the 7 geographical isolates of *C. reinhardtii *employed in this study (our unpublished data). For a genome rich in intergenic DNA this value is consistent with what one might expect under the mutational-burden hypothesis.

### Novel mitochondrial introns

An unforeseen consequence of this study is the discovery of 4 previously unreported *C. reinhardtii *mitochondrial introns, two of which (the L5- and L7-introns) are unusual. Although a detailed description of these introns is beyond the scope of this paper, they each contain a characteristic that is notable: 1) the L5-intron carries a 35 nt portion of the L5-rRNA-coding module within the 5 -end of its DNA sequence – this is an unprecedented feature for a mitochondrial intron. And 2) the insertion site of the L7-intron in relation to the *C. reinhardtii *LSU-rRNA secondary-structure model of Boer and Gray [21] corresponds to domain III (Figure 3); we believe this to be the first example (for any taxon) of an intron found in domain III of the LSU rRNA.

The discovery of 4 new optional introns in the *C. reinhardtii *mtDNA does not alter the notion that in *C. reinhardtii *the mitochondrial compartment is significantly more compact than the nuclear compartment: the mitochondrial genome of *C. reinhardtii *PA-1, the isolate with the most introns, is still ~67% coding compared to < 20% for the nuclear genome.

## Conclusion

The main objective of this study was to investigate genome compactness from a population-genetic perspective and, in doing so, test a contemporary hypothesis regarding the origins of genome architecture – the mutational-burden hypothesis. Our findings may not appear to be in full agreement with the mutational-burden hypothesis, we found ~4 times more nucleotide diversity in the nuclear compartment of *C. reinhardtii *relative to the mitochondrial compartment. However, when the 2*N*_*g*_μ-threshold for the proliferation of intronic and intergenic DNA is considered, we conclude that introns impose a greater burden on *C. reinhardtii *mtDNA and suggest that the intergenic regions of this genome do so as well. Overall, we cannot reject the mutational-burden hypothesis.

## Methods

### Strains, culture conditions, and DNA and RNA extractions

All of the *C. reinhardtii *strains employed in this study were obtained from the Chlamydomonas Center at Duke University in July of 2006, with the exception to *C. reinhardtii *CC-277, which was obtained from the same source in 1991. Clonal cultures of each strain were prepared from a single vegetative colony recovered on agar medium [20]. For each of the seven strains, total genomic DNA was extracted using the DNeasy Plant Mini Kit (Qiagen, Germantown, MD), and total RNA was extracted using the RNeasy Plant Mini Kit (Qiagen).

### Strain confirmation

To verify that the 7 *C. reinhardtii *isolates had been assigned the correct strain numbers we compared our sequences of the *ypt4*-VI, *ypt4*-VII, and *act-*VIII introns for each isolate to those obtained in other studies [16-18] from the corresponding isolates. The results confirmed that the 7 *C. reinhardtii *isolates employed here are the same as those used in previous reports.

### Amplification and sequencing of genetic loci

A PCR-based approach was used to amplify the mtDNA and the nuclear loci examined in this study. PCR experiments were performed in High Fidelity Platinum SuperMix (Invitrogen, Carlsbad, CA) using total genomic DNA as the template. Reverse-transcriptase (RT) PCR reactions were performed with the SuperScript III One-Step RT-PCR System (Invitrogen) following the manufacturer's protocol. PCR and RT-PCR products were purified using the QIAquick PCR Purification Kit (Qiagen). The purified products were sequenced on both strands at the Macrogen sequencing facility, Rockville, MD, USA.

### *C. reinhardtii *strain CC-503

The complete mitochondrial genome of *C. reinhardtii *strain CC-503 (cw92 *mt*^+^) was obtained by collecting and assembling mtDNA sequences generated from the *C. reinhardtii *nuclear genome sequencing project [19]. These sequences were acquired by blasting the complete *C. reinhardtii *mitochondrial genome against the following databases at the United States Department of Energy Joint Genome Institute (DOE JGI): *C. reinhardtii *v3.1-unplaced reads and *C. reinhardtii *v3.1-bonus scaffolds. Blast hits showing > 99% similarity to *C. reinhardtii *mtDNA were downloaded and assembled. All mitochondrial hits were subsequently checked against the draft nuclear genome of *C. reinhardtii *(v3.0 unmasked assembly) to insure that no nuclear mitochondrial DNA sequences (NUMTs) were collected. Our assembly of the downloaded mtDNA sequences contains over 500 chromatogram reads and gives a complete *C. reinhardtii *CC-503 mitochondrial genome with 15-fold coverage.

### Sequence analyses

To ensure that the 7 nuclear-encoded genes employed in this study are present only once in the *C. reinhardtii *nuclear genome we blasted each of the 7 sequences against the *C. reinhardtii *draft nuclear genome [19]. All 7 genes returned a single hit, which is consistent with the hypothesis that these genes are present in single copies in the *C. reinhardtii *nuclear genome. The mitochondrial sequences obtained from each of the seven strains were also blasted against the *C. reinhardtii *draft nuclear genome to confirm that they are not NUMTs: the blast results suggest that there are very few copies of mitochondrial sequences in the nuclear compartment and the few that are present are highly degenerate; therefore, we are confident that none of the mtDNA sequences presented in this study are NUMTs. The mitochondrial introns of *cox1 *and their secondary structures were identified using RNAweasel [34]. DnaSP 4.0 [39] was used for calculating all measures of genetic diversity. Nucleotide diversity and its standard deviation were calculated using equation 10.6 and 10.3 of Nei [40], respectively. Theta was calculated using equation 3 of Tajima [32]. The MacDonald-Kreitman test [26] and Tajima's D test [24] were performed in DnaSP.

### Accession numbers

Nucleotide-sequence accession numbers for the sequences that were employed in this study are shown in Table 4.

**Table 4:** Genbank accession numbers of the *Chlamydomonas reinhardtii *sequences employed in this study.

Strain	**mtDNA**^a^	***CBLP***^a^	***PETC***^a^	***PDK***^a^	***MAT3***^a^	***SFA***^a^	***ACTIN*-VIII**^b^	***YPT4*-VI**^b^	***YPT4*-VII**^b^
CC-277^c^	EU306622	EU306630	EU306651	EU306644	EU306632	EU306658	D50838	U13167	U13167
CC-1373	EU306617	EU306625	EU306646	EU306639	EU306633	EU306653	U70571	U55911	U55912
CC-1952	EU306621	EU306626	EU306647	EU306640	EU306634	EU306654	U70563	U55893	U55894
CC-2342	EU306620	EU306627	EU306648	EU306641	EU306635	EU306655	U70569	U55905	U55906
CC-2343	EU306623	EU306628	EU306649	EU306642	EU306636	EU306656	U70561	U55889	U55890
CC-2344	EU306619	EU306629	EU306650	EU306643	EU306637	EU306657	U70562	U55891	U55892
CC-2931	EU306618	EU306624	EU306645	EU306638	EU306631	EU306652	U70568	U55901	U55902

^a ^Present study.

^b ^Sequences from [18] except for D50838, which is from [17], and U13167, which is from [16].

^c ^The mitochondrial genome from this strain was shown to be identical to that of *C. reinhardtii *CC-503.

## Authors' contributions

DRS carried out the molecular studies, data analyses, and drafted the manuscript. RWL helped in interpreting the data and revising the manuscript. Both DRS and RWL have read and approved the final version of this manuscript.
